# Neurosurgical management of anterior meningo-encephaloceles about 60 cases

**DOI:** 10.11604/pamj.2015.21.215.6313

**Published:** 2015-07-24

**Authors:** Loubna Rifi, Amina Barkat, Abdeslam El Khamlichi, Malek Boulaadas, Abdessamad El Ouahabi

**Affiliations:** 1 Department of Neurosurgery, National Centre of Rehabilitation and Neurosciences, Rabat, Morocco; 2 Mohamed V University Rabat, Morocco; 3 Medical Department of Neonatology Reanimation, The Reference National Centre of Neonatology and Nutrition of Mother And Child, Sick Child Hospital CHU De Rabat, Morocco; 4 Department of Maxillo-Facial Surgery And Otolaryngology, Hôpital des Spécialités ONO, CHU- Rabat, Morocco

**Keywords:** Anterior meningo-encephaloceles, sincipital, craniofacial surgery, and common teratogen

## Abstract

Anterior meningo-encephaloceles (AME) are congenital malformations characterized by herniation of brain tissue and meninges through a defect in the cranium, in frontal, orbital, nasal and ethmoidal regions. The management of this complex congenital malformation is controversial according to whether use, an intracranial, extra-cranial or combined approach. This is the first largest series published in Africa, in which we present our experience in the operative management of AME; we share our recommendation in technical consideration for surgical approach with review of the literature. All patients beneficed of neuro-radiological investigations including Plan X rays, Spiral Three dimensional CT scan and MRI. Ophthalmologic and maxillo-facial evaluations were done in all the cases. MEA are surgically approached in various ways, mainly on the basis of its location and type, by cranio-facial approach in one-step, or in two stages by intracranial approach followed by facial approach, only by cranial approach or facial approach. The surgical results were evaluated in the follow up on the basis of disappearance of cranio-facial tumefaction with correction of hypertelorism. 60 children with AME were treated in our department between January 1992 and December 2012. The mean age at time of surgery was 14 months (20 days to 18 years) with slight men predominance (28 females/32 males). Cranio-facial team operated 21 patients, 16 were operated in two stages by intracranial approach followed by facial approach, 20 cases beneficed the neurosurgical approach and three only the facial approach Some post operative complications were observed: 2 cases of post operative hydrocephalus underwent shunt; CSF fistulas in three cases cured by spinal drainage, one death due to per operative hypothermia, 3 cases of recurrence how needed second surgery. After mean follow up for 80 months (1 year to 19 years) theses techniques permitted a good cosmetics results in 42 cases, average cosmetics results in 8 cases, poor results in 5 cases and worse cosmetics results in 4 cases, The AME are rare conditions we used the multiples approach first intracranial approach followed by facial approach, but after 1998 we used one-step correction by combined approach, only cranial approach when needed or facial correction.

## Introduction

In 1855, Finnish physician Karl Benedikt Mesterton described a congenital malformation characterized by the herniation of the mining's and cerebral tissue through a defect in the anterior cranium. He classified this pathological entity, now known as fronto-ethmoidal meningo-encephaloceles (MEC) into three types according to its location: nasofrontal, nasoethmoidal and naso-orbital. In the same time spring in 1854 wrote an excellent monograph on the subject, which was probably the first major work about this condition. He made a distinction between a meningocele and cerebral hernia. It is an endemic deformity found more commonly in the eastern hemisphere. In Southeast Asia (Thailand, Burma and Cambodia) and parts of India, Russia and Africa, the Highest incidence reported in 13500 live births, this lesions often affect poor rural children in developing countries [1] in contrast North America, Japan, Hong Kong and the western state of Europe the incidence is as low as 1/35000 live births [2]. Its aetiologies are still debated and poorly understood. Many papers have been published on AME treatment with modern medical infrastructures and endoscopy for the transethmoidal encephaloceles. In our department we have operated them in different techniques with cranial or facial approaches depending on the type of the meningo- encephalocele. This is the first largest series published in Africa, in which we present our experience in the operative management of MEA; we share our recommendation in technical consideration for surgical approach with review of the literature.

## Methods

All of our patients underwent a clinical evaluation including family history, the follow of pregnancy and delivery, cranio-facial examination and ophthalmologic study. Plain X rays, CT scan and tridimensional computed tomography and MRI study were done for all the patients. We have classified our cases according the classification of Swanwela et al [2] Rosenfeld et al [3] into fronto-ethmoidal and basal meningo-encephaloceles. Four cases of transethmoidal MEC were referred by ORL after trans-nasal biopsy and CSF leakage, the diagnosis was confirmed by MRI study in this cases. All patients had ophthalmological, ORL examination, to explore others systemic malformations we complete by echocardiography and abdominal echography. Before each operation for the fronto-ethmoidal meningo-encephaloceles, the photographs of the face in different incidences were obtained. The postoperative photographs were tacked and also three months after surgery and yearly schedule.

**Figure 1:**
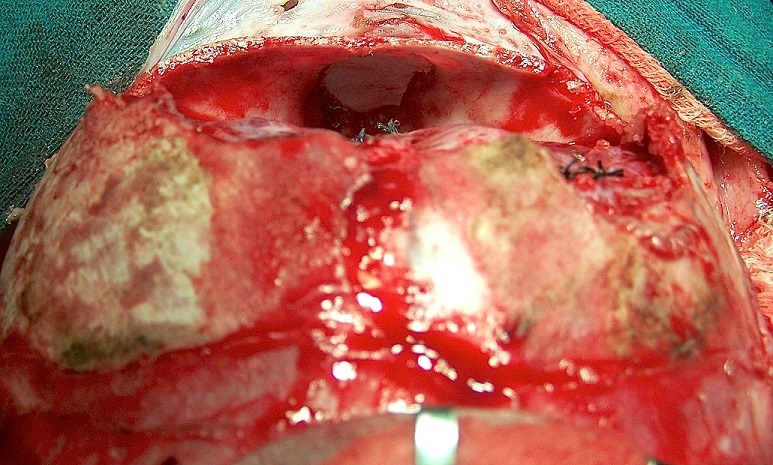
Cranial approach for AME

**Surgical technique:** All operations were performed with the patient under general anaesthesia. A standard neurosurgical procedure was used to treat AME. After shaving the patient's head, the skull and face were prepared with Betadine and draped. The adrenaline solution was infiltrated along the bicoronal incision line. A frontal galeal flap was elevated, and the dissection continued to the supra-orbital rim. Both supraorbital nerves were dissected from their osseous notch and preserved but when we found the osseous canal we have some difficulties to preserve these nerves. Tissues on the glabellas were then elevated until the rim of the defect made by the herniation was reached. A frontal bone flap was then outlined, with two frontal bur holes and two bur holes at the super medial part of the orbit. The electric drills or giggly saw were used to connect the holes and the rim of the defect to raise the flap. Once the bone flap was elevated and removed, the cerebral hernia was exposed at its base and could then be manipulated to allow ligature of the base (Figure 1). The hernia dissection is the most difficult part of the procedure because the Dura mater in this area in children is thin and adherent to the bone; dissection must be careful to avoid dural tears. The dura was sutured after total resection of the hernia content, the sutures of the dura was usually reinforced with a patch of pericranium and fibrin glue. A piece of cortical bone was used for reconstitution of the glabella region and for the closure of the bony defect in the ethmoidal region; we had never use the osteosynthesis. Medial canthopexy was achieved in all patients. We have used several techniques according the type of the meningo-encephaloceles.

**The first surgical technique:** In the patient with large herniation covered by a large expansion of the facial skin, a combined trans-cranial and facial procedure was used. The amount of facial surgery varied with the size of the mass and associated abnormalities. Redundant Para nasal soft tissue was removed in an inverted Y-shape, and the nasal tip was hitched up to the nasal bones with no absorbable sutures Outlining incision and resecting the skin, to obtain a minimal residual scar designed to fall into the medial canthal area extending down to the naso-labial fold or up to the midline of the forehead. The meningo-encephalocele was then totally or partially resected so that facial contours would be normalized (Figure 1).

**Second surgical approach:** In the patients with minimal soft tissue expansion into the face the meningo-encephalocele was removed through the craniotomy alone; nasal and/or orbital corrections were made afterwards. In orbital encephaloceles we have do only the cranial approach.

**Figure 2:**
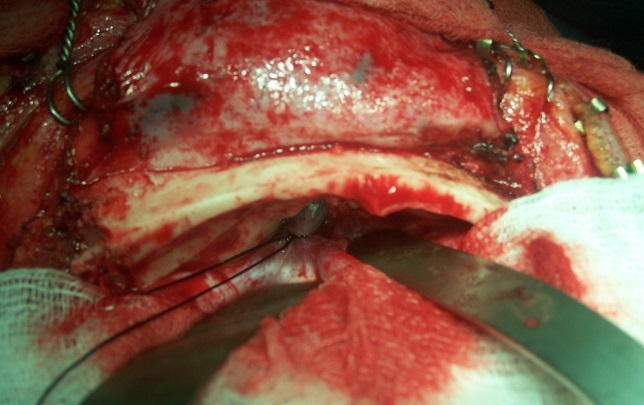
Cranial approach for trans-ethmoidal meningoencephalocel

**Third surgical approach:** In the transethmoidal meningo-encephaloceles the bicoronal incision was made. The pericranium and musculo-fascial flaps were prepared for anterior fossa reconstruction. After extradural dissection of the frontal base, the neck of the meningo-encephaloceles was identified passing downward into the nasal cavity through a defect in the side of the cribriform plate, and the stalk of the meningo-encephalocele was amputated (Figure 2). The Dural defect was then water tightly closed with the pericranial flap. The bone defect of the frontal fossa was covered by bone graft taken from the superior part of the frontal bone and reinforced by frontal pericranium. A formal hypertelorism correction was not performed routinely. When indicated, the medial canthal ligaments and overlying soft tissues were relocated. Ophthalmologists repaired pre-existing strabismus 2 to 3 months after the primary repair.

**Figure 3:**
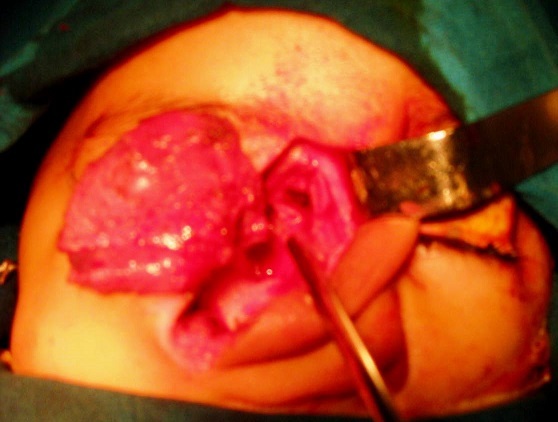
Facial approach for fronto-nasal meaning-encephalocel

**The facial approach** Facial incision is routinely made for accessing the facial structures, removing the mass as well as resecting the covering skin which is usually expanded and redundant. This study demonstrates the possibility of doing definitive surgery with minimal facial incision and bone defect reconstruction with the alloplastic material (Figure 3).

**Morphologic evaluation:** We classified our global aesthetic results in three categories: a poor result means that the postoperative facial appearance of patient was still incompatible with a normal social life, without improvement of aspect; an average result mean that the patient's facial appearance was improved but with some abnormalities; a good result meant the facial appearance was normal with a few scars.

**Follow up of the patients:** All of our patients had ten days hospitalisation; they were put on antiepileptic medications (Valproic acid) antalgic and antipyretic drugs. For the intellectual development we follow the child adaptation in school and with the success in their exam until the adolescence.

## Results

It's the retrospective study about 60 cases of AME treated between 1992 and 2012 in our department. The number of cases has remained similar over the last twenty years ranging from two to three cases on the average per years. This number is higher than the posterior meningo-encephaloceles how is 27cases received in the same period.

**Origin of patients:** 50 children had rural origin and they came from different regions of Morocco without any ethnic origin, and in 22 cases received before 1998 the pregnancy and delivery hadn't been following up.

**Clinical findings:** There were 32 males and 28 females patients ranging in age from 20 days to 18 years (average age of 14 months) only 4 trans-ethmoidal meningo-encephaloceles and one fronto-orbital meningo-encephalocele were diagnosed over the age of 5 years. Physical examination of the encephaloceles revealed skin covering in all patients with very thin skin in two babies aged respectively 20 days and 50 days. In addition 17 patients showed stigmata of the sack, such scarring, pigmentations, some abnormal hair or discoloured spots. 21 patients had eye displacement, 13 showed the hypertelorism and the growth of the head circumference was present in 5 cases. The long nose deformity showed in 5 cases, the cornea dystrophy noted in two cases, one case of exophthalmia, 12 patients had dacryocystis. 4 cases were received with Rhinorrhoea after ORL biopsy and one 18 years old girl with orbital encephaloceles was received with ocular leakage of CSF after ophthalmologic surgery. Psychological retardation was found in 3 patients (Table 1).

**Table 1:** Encephaloceles classification in 60 patients

	Classification	Cases
Frontoethmoidal (50)	Nasofrontal	25
	Nasoethmoidal	16
	Naso-orbital	9
Orbital encephaloceles		6
Trans-ethmoidal		4

### Radiological examination

Based on clinical examination and radiological finding, Plain X rays, CT scan and MRI examinations the 53 frontoethmoidal encephaloceles could be classified into: 25 nasofrontal, 7 nasoethmoidal unilateral, 8 nasoethmoidal bilateral, 9 naso-orbital, 4 naso-ethmoido-orbital and 4 trans-ethmoidal (Table 2). All patients showed an internal median skull defect. In 25 patients this skull defect was situated at the site of the foramen caecum. In the 10 with naso-frontal encephaloceles the skull defect was located just superior to the foramen caecum. In 7 patients the defect was located just lateral of the foramen caecum and in 5 patients with trans-ethmoidal encephaloceles the defect was located in the cribriform plates. The size of the skull defect ranged between 0.5 x 0.5 to 4.0 x 3.5 cm. the herniated mass was related to one frontal lobe in 35 cases and to both frontal lobes in 22 cases. In three cases it was only meningocels treated by facial approach. Additional intracranial anomalies were seen in 34 patients with 26 patients with normal brain in CT scan and MRI. These additional anomalies were well interpreted with MRI. A list of these findings is presented in (Table 3).

**Table 2:** Clinics futures in 60 cases of anterior meningo-encephaloceles

Conditions	Number of cases
Epiphora (bilat or unilat)	12
Canthal dystopia	18
Strabismus	6
Seizures	2
Microphthalmia (bilat or unilat)	5
Cognitive delay	3
Clinical hydrocephalus	4
Corneal scar	2
Hypotonic	2
Rhinorrhoeas	4

**Table 3:** CT scan with 3 dimensional reconstruction / MRI findings in 60 cases

CT scan/MRI finding	Cases
Skull defect	17
Disjunction of sutures	5
Orbital rim enlargement	5
Nasopharyngeal mass	4
Hydrocephalus	4
Porencephalic cavity	1
Agenesis of corpus collusum	5
Arachnoids cyst	7
Schizencephaly	1

### All our patients underwent surgery

**Figure 4:**
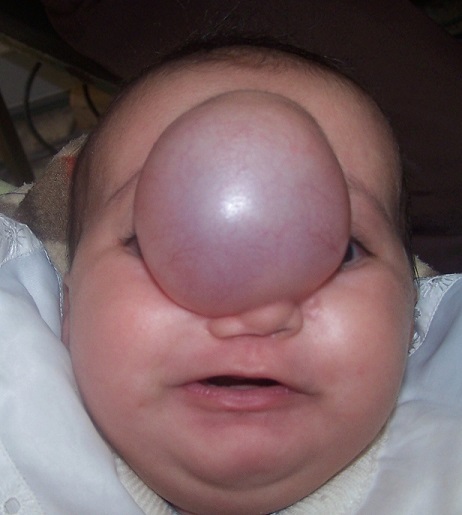
50 days old girl with front-nasal meaning-encephalocele treated by combined approach

All our patients underwent surgery: the neurosurgeon and the maxillofacial surgeon operated 21 patients in the same surgical time (Figure 1); 20 patients were operated only by cranial approach, including 4 patients with trans-ethmoidal meningo-encephalocele, 5 patients with orbital meningo-encephalocele and 11 patients who's the facial masse was medium size with good quality of skin (the second approach); 16 of children were operated by trans-cranial approach followed by facial approach, with surgery in two step (Figure 3); 3 nasoethmoidal meningocels with a small skull defect were cured only by facial approach (Figure 4). 36 patients were operated only by trans cranial approach the surgical technique was identical and the different steps of the intervention were respected, the encephalocele was removed, the watertight closure of the dura was made and the bony defect was obliterated by bone graft taken from the frontal bone, 16 of these patients were operated at the age of five years old by maxillofacial team when the facial anomalies persisted and the child had been schooled. 20 patients did not have a facial aesthetic anomaly. The neurosurgeon and the maxillo-facial surgeon operated 21 patients, with a redundant Para-nasal soft tissue in the same time. The trans-cranial approach was performed at first followed by the resection of the Para-nasals soft tissue. Three nasoethmoidal meningo-encephaloceles with a small defect in the skull were removed only by facial approach, the Para-nasal soft tissue was removed and the collet of the MECs was dissected, sutured and the closure reinforced by surgical glue, the bony defect was obliterated by a bony graft taken from the iliac crest.

**Hydrocephalus:** In our series 4 patients with symptomatic hydrocephalus underwent shunt before craniofacial surgery, and 2 children developed hydrocephalus after craniofacial repair required shunt 8 days and 15 days post operatively.

### Immediate postoperative complications of the patients

Postoperative CSF leak under the skin or through the facial scar was observed in 4 patients. Lumbar puncture and compressive head dressing treated these leaks. Two children developed hydrocephalus how underwent shunt. Despite of anticonvulsant therapy two children had recurrent seizures how were stabilized under Carbamazepine.

**The perioperative death:** There was one death in this series the new born baby 20 days old with ulcerated and leaking MECs was death preoperatively because of hypothermia, and anaesthesia procedure.

### The complications observed at the follow up

One case of trans-ethmoidal MECs had developed three years after surgery meningitis and the MRI study showed the complete resorption of the bony graft and recurrence of the meningocels. He underwent second surgery by trans cranial approach the defect was found the dura matter was very thin in the border of the dura graft. The fascia lata graft was used to reinforce the watertight closure of the dura. Two others naso-frontal MECs had relapsed after two and three months post operatively with recurrence of the swelling in the site of the malformation and despite lumbar picture and spinal drainage we had to do second surgery and we have found fistula.

### The follow up cosmetic and social results

The follow up period ranged from one year to 20 years with mean of six years, good psychomotor development in 56 cases but one case with shizencephalyhad delayed psychomotor development the two other cases with Porencephalic cavities and agenesis of corpus collusum had delayed school efficiency. Pre, per and after each operation, photographs of the face were obtained; three months post operatively and after one year, and each two years. To appreciate the cosmetic results we use the patients or parents’ self-evaluation, the surgeons and all the team of department evaluation (Table 4).

**Table 4:** Cosmetic results in 59 surgically treated patients

Cosmetics results	Surgical team evaluation	Patients or parents evaluation
Good	42	45
Average	08	09
Poor	05	03

### The illustrative cases

**Figure 5:**
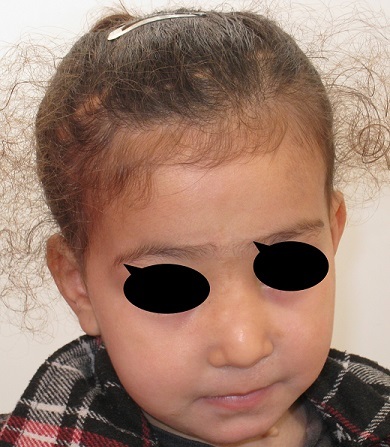
The same girl three years later

**Figure 6:**
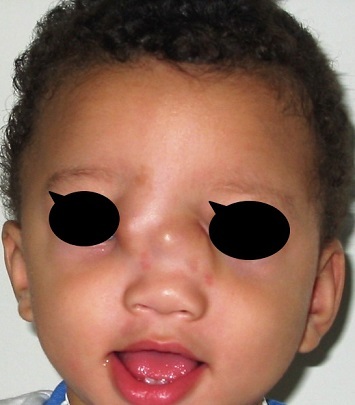
The 9 months old boy with fronto-nasal meaning-encephalocele operated first with cranial approach, and facial approach 6 years later

**Figure 7:**
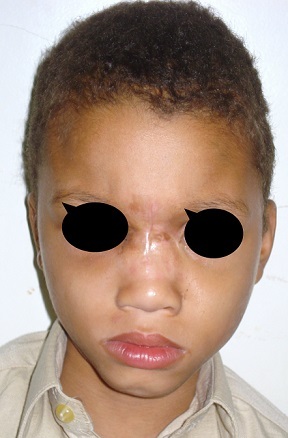
The same boy after facial surgery

First case: 50 days girl who presented a fronto - nasal meningocele has been operated by neurosurgeons and the maxillofacial procedure simultaneously (Figure 4) shows the preoperative appearance and Figure 5 shows the appearance of the child 3 years later with a good result Second case: the 9 months old boy with fronto-nasal meningo-encephalocele (Figure 6). Operated first with cranial approach, and facial approach 6 years later. The same boy after facial surgery (Figure 7) with average cosmetics results.

## Discussion

Despite the reduction of neural tube defects (NTD) in the western countries, in Morocco NTDs are still the major problems for the neurosurgeon especially for paediatric neurosurgeons. The department of neurosurgery in the hôpital des spécialités is the reference centre in Morocco and the first centre in Rabat for paediatric neurosurgery. We get large number of patients with NTD including encephaloceles. The anterior meningo-encephaloceles are rare. 60 cases were treated in our department over the period of 20 years and this is the largest series of AME published in Africa. This series are higher than those recorded by several authors in Africa. Indeed, Amadi et al [4] presented the retrospective study of children with encephalocele admitted from January 2007 to December 2009, 17 cases were seen over this period. 12 presented as frontal encephalocele while 5 were occipital. However, Kalungo et al [5] noted a series of eight anterior encephaloceles. But in other studies Aditiloye et al [6] 10-year reviewed of infants with encephalocele was carried out in a multicentre teaching hospital, out of 23,438 infants seen within the period, only 12 cases of encephalocele were seen, giving an incidence of 0.5/1000 births. Nine (75%) of patients had occipital encephalocele, two cases (16.7%) occipito-parietal and one cases (8.3%) fronto-nasal. Smit et al [7] detected during a period of 4.5 years, 14 patients with frontoethmoidal meningo-encephaloceles were treated. Most patients came from Northern Namibia. A retrospective study of 9 cases of fronto-ethmoidal encephaloceles was presented By Ba MC et al [8]. The cases have been gathered from the files of Dakar University Neurosurgical Unit. Frontoethmoidal type is the commonest type reported in the literature [2, 9] we had treated 56 cases of frontoethmoidal MECS with 25 naso-frontal, 16 naso-ethmoidal, 9 naso-orbital and six orbital (Table 1). The basal encephaloceles are rare entity; Gerhardt et al [10] classified them on trans-ethmoidal, spheno-ethmoidal, spheno-orbital, spheno-maxillary and trans-sphenoidal. We have treated 4 cases of trans-ethmoidal MEC.

### Geographical distribution, incidence and aetiology

Meningo-encephaloceles are not a homogeneous disease entity, occipital MECs predominate in Europe and north America whereas sincipital or AME are common in southeast Asia such Cambodia, Thailand, Burma, Malaysia and Indonesia [1, 11, 12]. Monteith et al [13] reported ethnic distinctions in New Zealand, with patients of Pacific Island descent having a much higher incidence of sincipital lesions (44%) than those of European origin. Suwanwela et al [2] reviewed the world's literature up to 1970, and also found a very high incidence of sincipital lesions reported from Russia (86%). In the same review, he reported relatively high incidences from South Africa (47%), and Morocco in northern Africa (39%). This condition is also found in India [14], and Australia in the aboriginal population, Mexico and Turkey. There have been few studies of the encephaloceles originating in sub-Saharan Africa. Suwanwela et al [2] uncovered a single small Nigerian series of encephaloceles, in which 19% of lesions were sincipital. Several more recent studies from the western African countries of Nigeria and Senegal have reported only 8%-12% of lesions as being sincipital, with the great majority being occipital. C.S.F.Smit et al [7] presented a series of 14 cases of frontoethmoidal meningo-encephaloceles originated from northern Namibia. In our department we have collected the sporadic cases of meningo-encephaloceles from divers rural and urban regions with ethnically divers groups. In the same period we have treated 60 anterior meningo-encephaloceles and only 27 cases of posterior meningo-encephaloceles. The cause of these lesions remains obscures and it's same to be sporadic in most cases [12]. The several aetiologies were suggested, genetic predisposition, parental consanguinity. In our series the consanguinity was reported in only 5 cases. The Vitamin B deficiency due to maternal malnutrition and drugs proposed by Suphapeetiporn K et al [15], but refuted by others. Furthermore it's evident for the majority of authors [1, 15] this disease has preferential occurrence in poor and rural people. In our series 70% of our patients had rural origin, which could also explain the very low prevalence of AME in developed western countries. Familial cases are very rare we haven't found any familial cases in our series. Oucheng et al. [1] demonstrated that the conception in the wet season might play a role by founding fungal and teratogen agent such Alfa-toxin or Ochratoxin. Wangikar et al [16] reported that the toxins may be present in the diet in higher concentration during the hot, humid months and when foodstuffs are stored for long periods in poorly ventilated rooms. Furthermore, they had demonstrated that the children from Thailand, where AME frequently occur, have high Alfa-toxin concentration in their secretion and tissues. Warf BC et al [17] in his study for encephaloceles in Uganda in which he compared the date of birth for all the patients and selected those born in rainy season months or dry season months. He concluded: there was not relationship between season of birth and number of sincipital meningo-encephaloceles versus others lesions. In our series we haven't found any correlation between the date of birth and the meningo-encephaloceles, but the majority of the children with this malformation are coming from the rural and poor regions of Morocco. Another factor also maybe involved in the development of encephaloceles is the deficiency of folic acid. Rowland CA et al [18] demonstrated that the prevalence for the encephaloceles hasn't change after fortification with folic acid, but the prevalence rate for Spina bifida had decreased significantly. Although the cause of encephaloceles remains unclear, it appears to involve a variety of genetic and environmental factors.

### Pathogenesis and natural history

There has been much speculation about the pathogenesis of AME. There are several hypotheses: The first hypothesis involves neural tissues herniating through a point of weakness in the facial skeleton. On the basis of the embryological studies proposed that the weak point exists between the frontal and the ethmoidal bones and the deficiency in this area may lead to MEC. David et al [19] suggested a blow out of the intracranial contents through a midline tunnel from the anterior cranial fossa into the facial skeleton. The second hypothesis proposes that delayed closure of the neural tube prevents normal union of facial bones. However, this condition is not classified in the category of neural defects. The admitted theories involve a local deficiency of the mesoderm in combination with abnormal adhesion of the neuro-derm into the surface of ectoderm. The natural history is variable among patients, neurological complications are uncommon in AME [2], but congenital brain anomalies are frequents in this pathologies. In our series, the Porencephalic cavities were present in 5 cases, the asymptomatic arachnoids cysts in four cases, schizencephaly in two cases and agenesis of the corpus collusum in 5 cases (Table 3) like the series of Smit et al [7], how had found 8 schizencephaly, 5 agenesis of corpus callosum. These anomalies have been attributed to teratogen exposure toward the end of the second gestational month. Ouchen et al [1] hadn't reported cerebral anomalies in his series of 200 consecutive cases. Facial malformations are almost universally present and include hypertelorism, median nasal fissure, broad nasal root, cleft lip or palate, median cleft face syndrome or cranium bifidum. Optic malformations including: anophthalmia, or microphtalmia, colobomas, retinal abnormalities, morning glory syndrome. In our series we have found cornea dystrophy in 2 cases, anophthalmia 1 case, exophthalmia 1 case and colobomas 2 cases. The majority of affected children are mentally normal; in the Oucheng et al. [1] series he reported that the malformations didn't grow with time but some authors noted that child malformation became progressively larger [2]. In our series 2 meningo-encephaloceles growths quickly after birth one had the ulcerated meningo-encephalocele and one other had preruptured meningocele, so they were operated urgently. Some authors recommend early surgical repair to reduce the pressure effect of the mass on facial growth. In our series only three new-born were operated urgently: the first at the age of 20 days old for ulcerated front-nasal meningo-encephalocele, the second for preruptured fronto-nasal meningocele at the age of 30 days old, and the third cases for trans-ethmoidal meningo-encephalocele at the age of 50 days old with nasal obstruction. Usually the diagnosis is obvious in the frontal meningo-encephaloceles especially if there is clinical or palpable cerebral pulsation. Although any mass in the area may mimic an encephalocele, the most common differential diagnosis is a dermoid cyst or lipoma. The haemangioma of the glabella, nasal, and orbital areas may also present confusion in diagnosis. We have operated one orbital dermoid cyst mimicking nasofrontal meningo-encephaloceles. All the cases beneficed IMR investigations, three-dimensional CT scan to evaluate the bone defect in various angles. Planning of surgery and the time of operation is very important in management of AME. Prolonged surgery, in one stage correction can lead to blood loss and hypothermia, which remains the two important intra-operative complications in the paediatric neurosurgery. One of the new-borns with ulcerated encephalocele was died by hypothermia after one stage repair of his fronto-nasal meningo-encephalocele it's unique dead in this series. The time recommended for surgery is 6 to 10 months old or when the baby body weight is around 6 kg, we have operated under the age of three months only the emergency cases. Presence of hydrocephalus is an important factor in management of encephaloceles, if not treated prior to definitive surgery the risk of postoperative CSF leak is very high. The mechanism of postoperative hydrocephalus remains unclear; some authors proposed the common explication: that the encephalocele contains a large part of the resorption mechanism of the cerebro-spinal fluid. So the hydrocephalus must be managed in the post- operative period for all encephaloceles. The majority of the authors discussed the post-operative shunting. Mahaptra et al. [14] 20 patients of 133 presented with significant hydrocephalus underwent VP shunt prior to craniofacial surgery. Oucheng et al., [1] demonstrated that the hydrocephalus wasn't an important issue in the treatment of AME, only 4 out of 200 children treated required shunt in the post operative course. David DJ et al [19] concluded: that the presence of hydrocephalus or ventricular enlargement does not necessarily cause problems or require treatment. In our series 4 patients with symptomatic hydrocephalus underwent shunt before craniofacial surgery, and 2 children who developed hydrocephalus after craniofacial repair required shunt 8 days and 15 days post operatively.

### Surgical issues

Surgical therapy for this malformation is a complex task, its primary aim being to close reliably the connection between the intra-dural and extra-dural space. The choice of the best surgical treatment for AME is still debated. Meticulous planning is necessary for this choice depending in the type and size of the encephaloceles and associated hydrocephalus. Most authors recommend the combined procedure [1, 12, 20], the intracranial approach seems essential to us because only this technique guarantees a secure closure of the dura, to remove the encephaloceles and to repair the skull defect. We have used several techniques: neurosurgeon and maxillo-facial team operated 20 patients, 17 cases were operated by craniotomy first followed by facial approach and 19 patients were operated only by craniotomy. 5 were cured by the facial approach. In the interventions using only facial approach by only latero-nasal incision the relapse are common, we used this technique in 5 meningocels with very little cranial defect under 1,5 cm and we haven't had any relapse. But for two cases with a large skull defect, operated in others departments had relapsed and they were transferred in our department, the cranial approach was performed without relapse. The HULA procedure [14, 20] separates the supraorbital region from the medial canthal bone segment to allow for independent movement. The supraorbital bar is lowered and the medial canthal bone segment is positioned postero-medially. It is also important to stress that although the medial canthal segment must be moved medially, hypertelorism isn't treating by this technique. Finally, the procedure stressed a low radix position for the nasal cranial bone graft. We haven't used this method because of the difficulties of dissecting dura matter and meningo-encephalocele and to have a good closure of dura. Each method had its own value in certain situations. We had used several techniques: Facial technique when the skull defect is small and when we have only a meningocele or small meningo-encephalocele. It's easier by paralateronasal approach the meningocels or meningo-encephalocele was dissected and closed in this neck by sutures; we reinforced closure by human glue and a little bone graft, tacked away in iliac crest when needed. The cranial approach was used for 38 cases with total removal for the meningo-encephalocele and its closure by pericranial graft, the bone defect was closed by bone graft, tacked from the outer table of the skull. This technique permitted a good results and ovoid the scar in the face for 19 patients. But for the 17 patients operated before 1998 the facial repair was performed 5 years after cranial approach. We prefer a combined procedure, which has 3 major advantages: It's allows a better fronto-ethmoidal meningo-encephalocele closure, the better telecanthus correction and also reduce a facial scar. All the authors emphasized the advantage of one stage correction.

### Post operatives issues

The postoperative complications like any skull base procedure; the CSF leak is the most frequent and dread complication [1, 14]. Two patients in our series had postoperative CSF leak treated by spinal drainage. Three factors explain CSF leak: The no watertight closure of dura, an inadequate reconstruction of the skull base and also trans-facial approach. The recommendations of all the authors: The cranial base reconstruction is fundamental with bone graft pericranial patch and surgical glue. The dura mater around the meningo-encephalocele is very thin and must be dissected meticulously in this procedure we recommend to found the thick dura and to do sutures between pericranial patch with dura and reinforced by surgical glue. The trans-facial approach can be avoided when the MEC is small and facial skin of good quality. We hadn't used Trans facial approach for 19 cases. The scars could be avoided by daily treatment. We had only one case that developed the scars healing with trans-facial approach, which was treated by local care. The recurrence can be manage by second surgery, three cases in our series, recurred: one trans-ethmoidal-meningo-encephaloceles, operated at the age of 50 days, presented meningitis at the age of three years and the CT scan and MRI showed the local recurrence with total resorption of the bone graft, he underwent second surgery by cranial approach. The two others fronto-ethmoidal-meningo-encephalocele had swelling in the facial scar, two months and six months post operatively, and despite the lumbar puncture followed by spinal drainage, the swelling had persisted, so we decided to do the second surgery by trans-cranial approach. The three cases had good outcome. We have good cosmetics results in 42 cases, medium in 8 cases, poor in 5 cases and worse in 4 cases (Table 4). All the patients had a long follow up over 5 years, only 7 cases hadn't schooling; one of them with schizencephaly had delayed psycho motor development. All others children had normal schooling. However the patients with medium or poor cosmetic results have needed some cosmetics surgery they were transferred in the maxillofacial department.

## Conclusion

We present our experience on 60 cases of anterior encephaloceles who is one of the largest series in Africa. This malformation can be detectable by antenatal ultra-sonography screening (16-18 weeks). Imaging workup by CT scan tomography and 3 dimensional CT scan and also MRI allows precise study of meningo-encephaloceles, and associated malformations. Pathogenesis, classification and terminology remain a matter of debate. Surgical repair should be performed as early as possible to ovoid progressive enlargement of this conditions but the caution is required for the anaesthetics difficulties for the new born. The collaboration between neurosurgeon and maxillo-facial surgeon and neuro-paediatric anaesthesiologist is fundamental in this disease.
